# Mercury poisoning in women and infants inhabiting the Gangetic plains of Bihar: risk assessment

**DOI:** 10.1186/s12889-025-22336-9

**Published:** 2025-04-04

**Authors:** Arun Kumar, Radhika Agarwal, Kanhaiya Kumar, Nirmal Kumar Chayal, Govind Kumar, Rajiv Kumar, Mohammad Ali, Abhinav Srivastava, Siddhant Aryal, Tejasvi Pandey, Kumar Sambhav Verma, Dhruv Kumar, Rahul Laxman Gajbhiye, Sameer Dhingra, Naresh Pothuraju, Ramalingam Peraman, Akhouri Bishwapriya, Ranbir Nandan, Ashok Sharma, Manisha Singh, Ashok Kumar Ghosh

**Affiliations:** 1 Mahavir Cancer Sansthan and Research Centre, Patna, Bihar, 801505 India; 2 Lovely Professional University, Phagwara, Punjab India; 3 Amity University, Jaipur, Rajasthan India; 4 Indian Institute of Technology- Bombay, Mumbai, Maharashtra India; 5 UPES, Dehradun, Uttarakhand India; 6 National Institute of Pharmaceutical Education and Research , Hajipur, Vaishali, Bihar India; 7 Geological Survey of India, Ranchi, Jharkhand India; 8 Department of Geology, Patna University, Patna, Bihar India; 9 All India Institute of Medical Sciences, New Delhi, India

**Keywords:** Mercury poisoning, Breastmilk, Lactating mothers, Infants, Geospatial mapping

## Abstract

Mercury is a persistent, bio-accumulative, and hazardous contaminant. When released into the environment, it accumulates in water sediments, converting it to poisonous methylmercury that enters the food chain. The present study was carried out in habitations from the 11 districts of Bihar (India). In the study, *n* = 224 lactating women and their infants *n* = 172 infants participated. After obtaining the written informed consent, their breast milk, urine, and blood were collected for mercury estimation. The breastmilk content was measured in *n* = 181 subjects, in which 74% women had their breastmilk higher than the WHO permissible limit (< 1.7 μg/L), while 26% of the women had their breast milk below the permissible limit. The blood mercury content showed that 19% subjects had mercury content above the permissible limit [20 μg/L]. In urine mercury estimation, 49% women had mercury content above the permissible limit [10 μg/L], while, 51% women had the mercury content below the permissible limit. In the child’s urine, 54% infants had their mercury content in urine above the permissible limit [10 μg/L] while 46% infants had content below the permissible limit. The study indicates that 20% of infants had the complete accumulation of mercury in their body which is highly toxic for them. However, the mercury content in the food (wheat) had the contamination with in the permissible limit [100 μg/kg]. There was a significant correlation found between the breastmilk and child’s urine and mother’s urine. The HQ study also correlates the mercury poisoning effect with 100% of the mother’s and 66% of the infants exceeding the limit of non-carcinogenic risk. The Monte Carlo and multivariate study correlates the high health risk in the studied population due to mercury poisoning. The entire study concludes that population inhabitation in the Gangetic plains of Bihar are exposed to mercury poisoning which may be due to geogenic or anthropogenic sources. But, the levels of mercury contamination above the permissible limit could lead to neurogenerative changes in the lactating mothers and their infants. To control the present problem medical intervention is immediately required.

**Supplementary Information:**

The online version contains supplementary material available at 10.1186/s12889-025-22336-9.

## Introduction

Mercury is classified as a dense metallic element, present in different states and can be detected in the natural surroundings as well as through human activities [1]. Mercury exists in various states, such as elemental and inorganic forms that are commonly encountered in occupational settings, as well as organic forms like methylmercury that are obtained through dietary consumption [2]. The levels of mercury fluctuate in terms of their harmful effects, influencing a range of bodily functions including the nervous, digestive, and immune systems, as well as affecting organs such as the lungs, kidneys, skin, and eyes [3]. Use of ayurvedic medicines and products, residential coal use, industrial processes, waste incineration, and mining, are major contributors to the release of mercury, despite its natural occurrence in the earth's crust [4]. Mercury may be also found in a variety of items, including batteries, measuring instruments, electric switches, dental amalgam, medications, and some light bulbs [5]. Mercury amalgam is used in almost all countries in dental health [6, 7]. In the medical field, substitutes for mercury-containing thermometers and sphygmomanometers are being investigated [8]. Burning coal also releases mercury and other hazardous air pollutants are released through industrial boilers, home stoves, and coal-fired power plants [9]. Around 24% of the world's mercury emissions, according to the 2018 Global Mercury Assessment, were caused by burning biomass, coal, and other fossil fuels [10]. Certain skin-lightening cosmetics include inorganic mercury, which is added is posing health risks to humans [5] Around the world, steps are being conducted to phase out items that contain mercury and lower the amounts of mercury in products [11, 12]. In the environment, mercury can be transformed by bacteria into methylmercury [13]. The World Health Organization has been closely monitoring the use of thiomersal, also known as ethyl mercury, as a preservative in various medications and vaccines as it is also a source of mercury poisoning [14, 15].

**Fig. 1:**
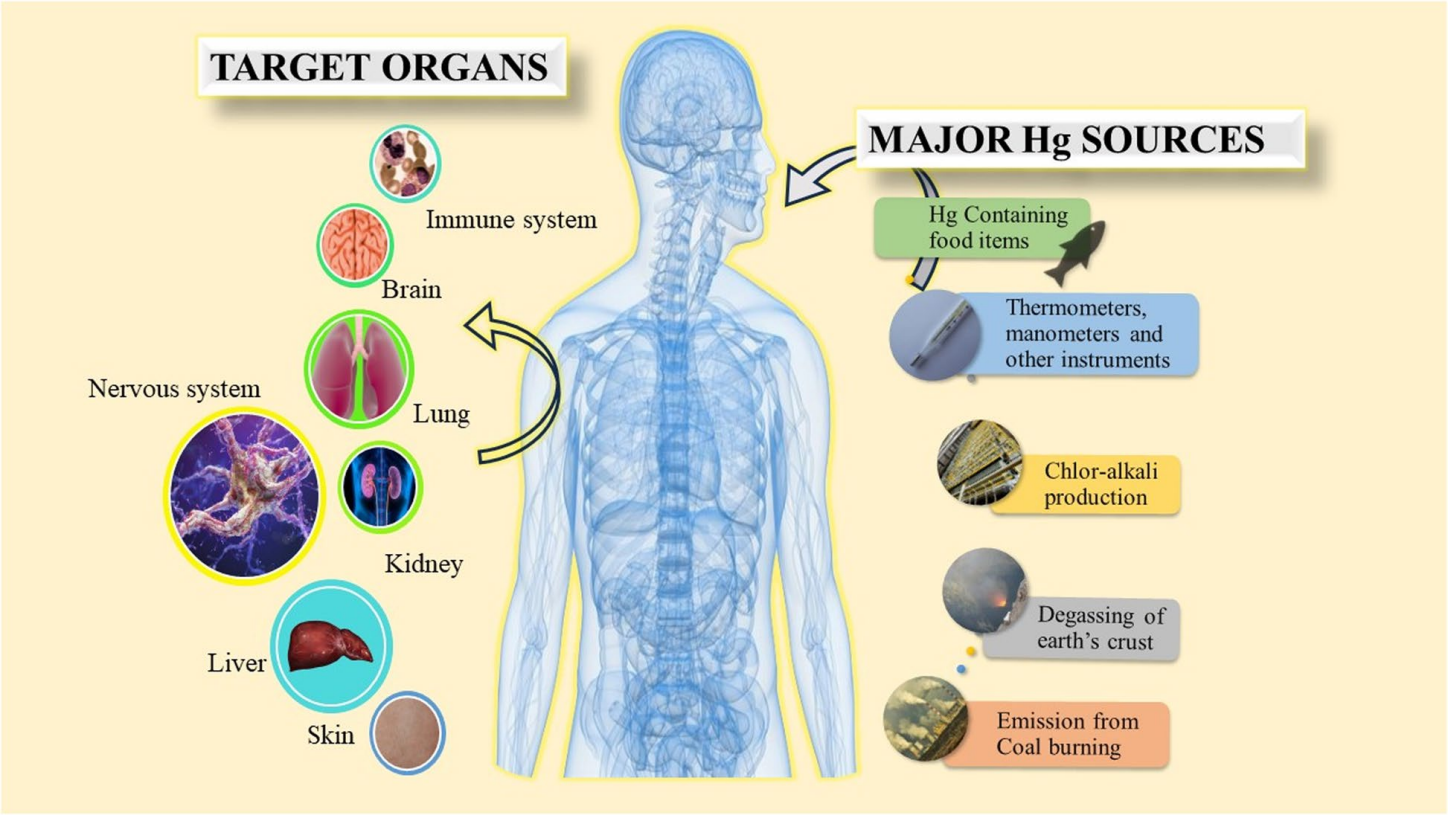
Showing effect of mercury contamination in human body

Methylmercury subsequently undergoes bioaccumulation, a process where an organism accumulates higher levels of the substance compared to its surrounding environment, within aquatic animals [16–18]. The system easily breaks downs methyl mercury and does not bio-magnify [19]. Exposure of methylmercury in the womb can result from a mother's consumption of fish and shellfish [20]. It can adversely affect a baby's growing brain and nervous system consequently, cognitive processes, memory retention, focus, linguistic abilities, as well as fine motor skills. It also causes deformities in visual-spatial aptitude due to methylmercury exposure during the prenatal stage [21] The exposure of mercury causes toxicity to vital organs of the human body causing health related problem in them (Figure 1). The mercury exposure can even lead to bioaccumulation in the exposed living organisms, including fish. And if this contaminated fish is consumed by the lactating mothers, then it can transfer this toxicity via breast milk to their nursing infants [22]. Breast milk is considered as the ideal food for infants, providing essential nutrients and immunity to the body [23]. However, the potential presence of contaminants such as mercury raises questions about the safety of breastfeeding [24].

By inhaling elemental and methyl mercury, both the central and peripheral nervous systems can get damaged which can lead to fatal outcomes and adversely affecting the immune, nervous, and digestive systems, along with the vital organs such as kidneys and lungs [14, 25–27]. Other toxicity symptoms such as tremors, sleeplessness, migraines, memory loss, neuromuscular effects, and cognitive and motor dysfunction are also caused due to its exposure [28]. For several years, workers exposed to an elemental mercury level in the air up to 20μg/m^3^ or more may have mild, subclinical indications of poisoning to their central nervous system [Fig. 1] [29].

In 2015, small-scale and artisanal mining contributed about 800 metric tons of mercury to air pollution, or 38% of the total global emissions [30–33]. There is a direct and substantial health risk associated with mercury poisoning for the 12 to 15 million workers in this industry worldwide [34]. A study in 2005 was conducted in Central region of India near a steel plant. This human breast milk and blood samples from 120 people associated to this steel plant were studied for hazardous metals (As, Pb, Mn, Hg, and Cd) contamination. For the comparison, blood and breast milk samples were also taken from the individuals residing outside of the steel plant. The study showed higher concentrations of these harmful components in their breast milk, blood samples [35]. Similar human studies on other heavy metals have been also reported [36]. Hence, the present study aims to estimate the mercury exposure in the subjects inhabiting in the Gangetic plains of Bihar (India) for the first time.

## Materials and methods

### Ethics approval

In accordance with the “WMA Declaration of Helsinki-Ethical Principles for Medical Research Involving Human Participants 2024” [37] and the Institutional Ethics Committee at the Rajendra Memorial Research Institute of Medical Sciences, under the Indian Council of Medical Research in Patna, Bihar, approved this study [IEC Letter No. RMRI/EC/24/2020, dated 26/09/2020]. Before participating, each person was informed of the study's goals, and written consent was obtained.

### Location of study

This study was conducted in selected districts of Bihar, India. The studied districts wee Bhojpur, Buxar, Saran, Patna, Vaishali, Samastipur, Darbhanga, Begusarai, Khagaria, Munger, and Nalanda. The study was conducted in the selected districts from October 2021 to October 2023.

### Selection of study subjects

The study focused on lactating mothers and their infants to determine mercury exposure in them. The study was conducted on *n* = 224 mothers, aged between 17–35 years. After obtaining the written consent, the studied subjects provided their breast milk, urine, and blood samples for mercury analysis. Additionally, 172 infants, aged between 0.26–30 months, their mothers provided urine samples of their infants for the study. An interview was also conducted with the participating mothers via a questionnaire related to breastfeeding durations, child growth related queries [Supplement-I].

### Sample collection

Each participating mother voluntarily provided 5 mL of their breast milk and blood, along with 50 mL of their urine samples. Moreover, the food (Wheat) samples 10 g was also collected for the contamination study from the same households. Samples were collected in respective sterilized containers and were stored at 2 °C–6 °C in a cool box, and were furthermore transferred to Mahavir Cancer Sansthan & Research Centre (MCSRC) in Patna, Bihar, India for further studies.

### Laboratory digestion process of breastmilk, blood and urine

To measure mercury levels, 0.5 mL of each sample were placed in a glass flask with 5 mL of nitric acid [HNO_3_] for overnight reaction. The following day, the samples were digested on a hotplate at 90 °C–120 °C until the volume reduced to 3 mL. A mixture of HNO_3_ and perchloric acid [HClO_4_] in a ratio of 6:1 was added, and the solution was further digested to 2 mL volume. After rinsing with 1% HNO_3_, the final volume was adjusted to 10 mL with distilled water which was thereafter filtered and stored in 30 mL glass vial for further analysis on ICP-MS [38].

### Estimation of food (Wheat) mercury concentration

The food samples (Wheat) (0.5g) were taken in 25mL conical flask and 5mL Conc. HNO_3_ was added and left for overnight reaction. The following day the samples were kept on water bath at 60 degrees centigrade for 2 hours. After the water bath digestion, the samples were allowed to cool at room temperature and then 2mL of HClO_4_ were added, and thereafter was heated on hotplate at 160^0^C for 5 minutes until the white dense fumes of HClO_4_ were released. The samples were then cooled at room temperature and the final volume was made by adding 10mL of demineralized water to the solution. The samples were then filtered with Whatman filter paper No. 41.

### Quality control of the chemical analyses

Adequate quality assurance and safety measures were taken to ensure the safety analytical methodology while performing sample analysis and handling the Inductive Coupled Plasma – Mass Spectroscopy (ICP-MS) instrument. The glassware employed for sample preparation was properly sterilized in order to prevent cross-contamination. All glassware and plasticware used in sample preparation and storage purposes were cleaned by soaking in 6% *v/v* HNO_3_ for 24 h and then followed by three cycles of washing with deionized water (Millipore Milli-Q, 18.2 MΩ cm − 1). The Teflon vessel used for sample digestion was cleaned by soaking in 2% HNO_3_ for 24 h, then rinsed with deionized Milli-Q water and dried in the dedicated hot air oven.

### Instrumentation and method conditions

Agilent 7850 instrument (USA) LC- ICP-MS was used to quantify mercury in blood sample extracts. The sample was introduced as a micro-mist using a peri-pump and auto-sampler SPS4. The optimal operating parameters are depicted in Table 1. For the testing procedure of Hg, the ICP-MS instrument condition was calibrated and validated as per the instrument's standard operating procedure. The argon plasma was generated to produce a temperature of 10000 K with 1550 kW of radio-frequency power. The plasma and He-gas flow were at 16.0 L/min and 4.3 mL/min, respectively. The argon plasma ionized the sample and then atomized it in the spray chamber. Repetitive washing with blank was used as a strategy to avoid carry-over. The ions ejected from the plasma, travelled across the instrument's interface, and eventually arrived at the collision cell's entrance. Here, helium gas was added to eliminate polyatomic interferences.

**Table 1:** Permissible limits of studied samples with references

Sample type	Permissible limit	Reference
Blood	< 20 µg/L	[40]
Breastmilk	< 1.7 µg/L	[41]
Urine	10 µg/L	[42]

### Calibration standards

A calibration blank (2% HNO_3_) was used to establish the calibration curve and determine detection limits (in ppb). A calibration standard solution containing 29-analyte (10 ppm) IMS-102; As, Se, Al, Cr, Mn, Ti, Sr, Co, Rb, Ag, Fe, Ni, Cs, Cu, Zn, Cd, Hg, Be, and Pb elements in the range of 0.1,0.2,0.3,0.5,1 and 2 ppm was prepared for calibration. The *r*^2^ value, slope, intercept, and BEC value are used to assess the suitability of the method.

### Correlation coefficient

The mercury levels in breast milk, maternal urine, and child urine were analyzed for correlations, and graphs were plotted using SPSS-25.0.

### Multivariate analysis

For multivariate analysis study, SPSS-20 was used to analyze through general linear model. For dependent variable study- child urine mercury and mother urine mercury values were used and breast milk as fixed factor.

### Geospatial analysis

Arc-GIS software [version 10.3.1] was used to map the mercury concentrations, with GPS coordinates of samples overlaid on a shapefile to visually represent mercury levels in blood, breast milk, maternal urine, and child urine.

### Human health risk assessment

The human health risk assessment is a technique through which the potential risk of carcinogenic as well as non-carcinogenic is evaluated. The health risk assessment has been calculated by using the formula given below.

$$\mathbf{C}\times \mathbf{I}\mathbf{R}\times \mathbf{E}\mathbf{F}\times \mathbf{E}\mathbf{D}/\mathbf{B}\mathbf{W}\times \mathbf{A}\mathbf{T}$$

where, C is the concentration of mercury, IR is the ingestion rate, EF is the exposed frequency and ED is the exposed duration, BW is the body weight and AT is the average time. The value of these variables is shown in Table 2, [39].

$$\mathbf{H}\mathbf{Q}=\mathbf{A}\mathbf{D}\mathbf{D}/\mathbf{R}\mathbf{f}\mathbf{D}$$

where,

**Table 2:** References of the health risk assessment studies

Parameters	Adult	References of Adult	Children	References of children
IR	159.9	[43]	0.741	[44, 45]
ED	30	[43]	0.25	[44, 46]
EF	350	[47, 48]	182	[45]
BW	71.7	[49]	7.4	[44, 50]
AT	22,365	[49]	45.5	[44, 45]
C[mg/L]		Present study		Present study
RfD	0.0003	[39]	0.0003	[39]
CSF		[51]		[51]

$$\text{HQ }=\text{ Hazard Quotient}$$

$$\text{ADD }=\text{ Average Daily Dose}$$

$$\mathrm{RfD}\;=\;\mathrm{Oral}\;\mathrm{Reference}\;\mathrm{Dose}\;\lbrack4\mathrm{\mu g}/\mathrm{Kg}/\mathrm{day}\rbrack$$

### Monte Carlo simulation by R studio software

Monte Carlo simulation is a probabilistic modelling technique which can used for the estimation risk including human health risk. It estimates uncertainties & variability in mercury exposed populations. This technique involves the generation of 10,000 random simulations based on the probability of the input data i.e. variables. According to National Academy of Science and USEPA, Monte Carlo simulation has been acknowledged as best for estimation of uncertainties in human health risk assessment [40].

Predictions of non-carcinogenic effects are possible when the resulting HQ value is more than 1, but predictions of no adverse health consequences from exposure are obtainable when the determined HQ is equal to or lower than 1.

### Statistical analysis

The statistical analysis was conducted using SPSS −25.0 and Graph Pad Prism 8.0. We graphed the concentrations of mercury in breastmilk, urine and blood [Table 1].

## Results

### Breastmilk mercury concentration

**Fig. 2:**
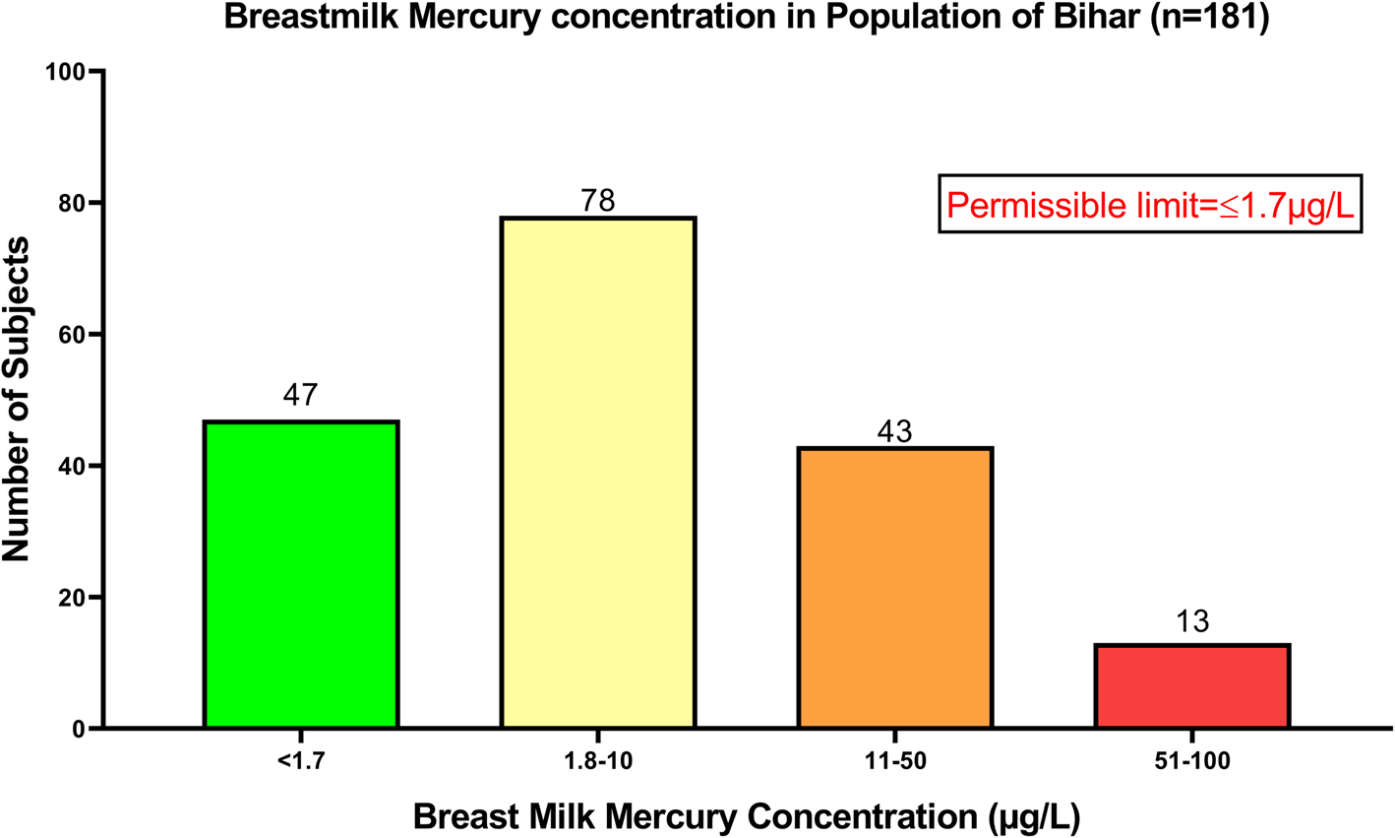
Graph showing mercury concentration in the breastmilk samples of the lactating mothers

*N* = 224 subjects were approached for providing breastmilk samples, out of which *n* = 43 subjects denied and only *n* = 181 subjects agreed to participate in the study. The results indicated that out of the total individuals (*n* = 181), *n* = 47 had breastmilk mercury concentrations below the permissible range, whereas *n* = 134 had levels higher than the permissible limit [< 1.7 µg/L]. There was a maximum of 61.91 µg/L of mercury recorded in breastmilk of one of the studied lactating mothers [Fig. 2].

### Blood mercury concentration

**Fig. 3:**
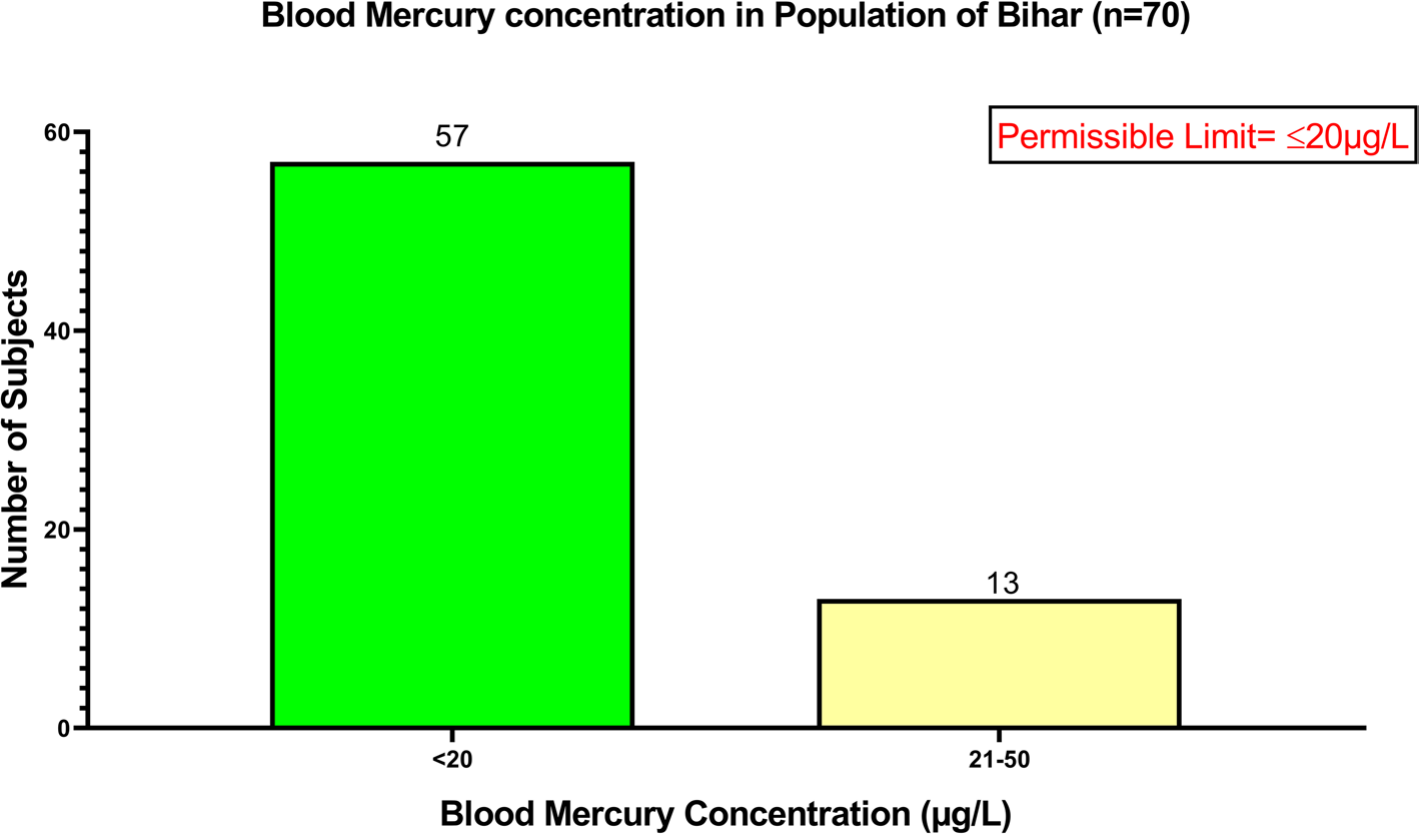
Graph showing mercury concentration in the blood samples of the lactating mothers

*N* = 224 subjects were approached for providing blood samples, out of which *n* = 154 subjects denied and only *n* = 70 subjects agreed to participate in the study. Therefore, *n* = 70 subjects had their blood mercury levels measured. Only *n* = 13 subjects had blood mercury levels greater than the WHO permissible limit [< 20 µg/L], with the highest value recorded as 38.35 µg/L, whereas *n* = 57 subjects had mercury concentrations below the permissible limit [Fig. 3.].

#### Mother’s urine mercury concentration

**Fig. 4:**
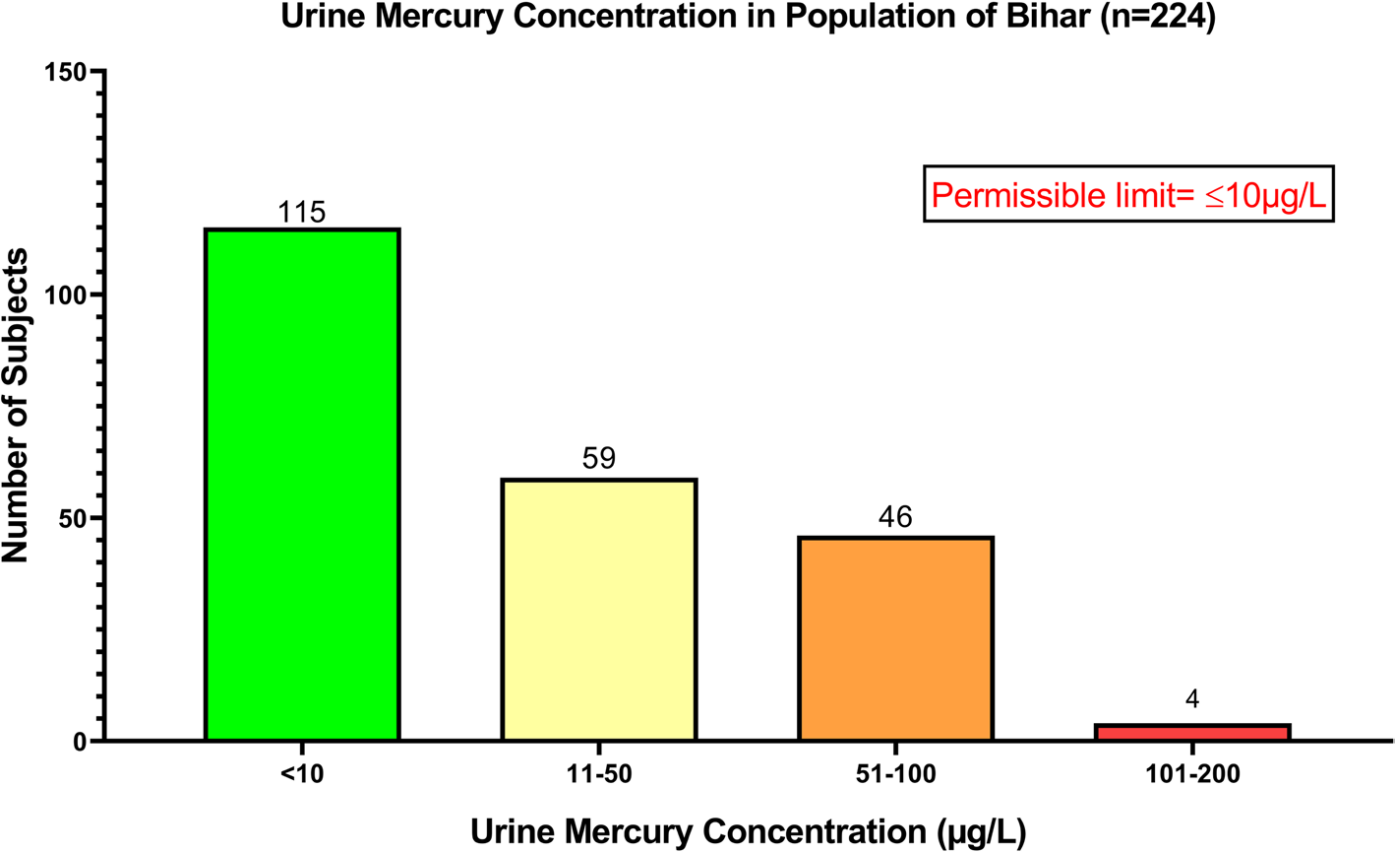
Graph showing mercury concentration in the urine samples of the lactating mothers

The urine mercury concentration was estimated in *n* = 224 subjects. Results indicated that *n* = 115 subjects had the mercury concentration in their urine below the permissible limit [10 µg/L], whereas *n* = 109 subjects had a concentration of mercury in their urine higher than the permissible limit. The urine had a maximum mercury concentration of 151.34 µg/L in one of the subject’s urine samples which was extremely very high [Fig. 4].

#### Child’s urine mercury concentration

**Fig. 5:**
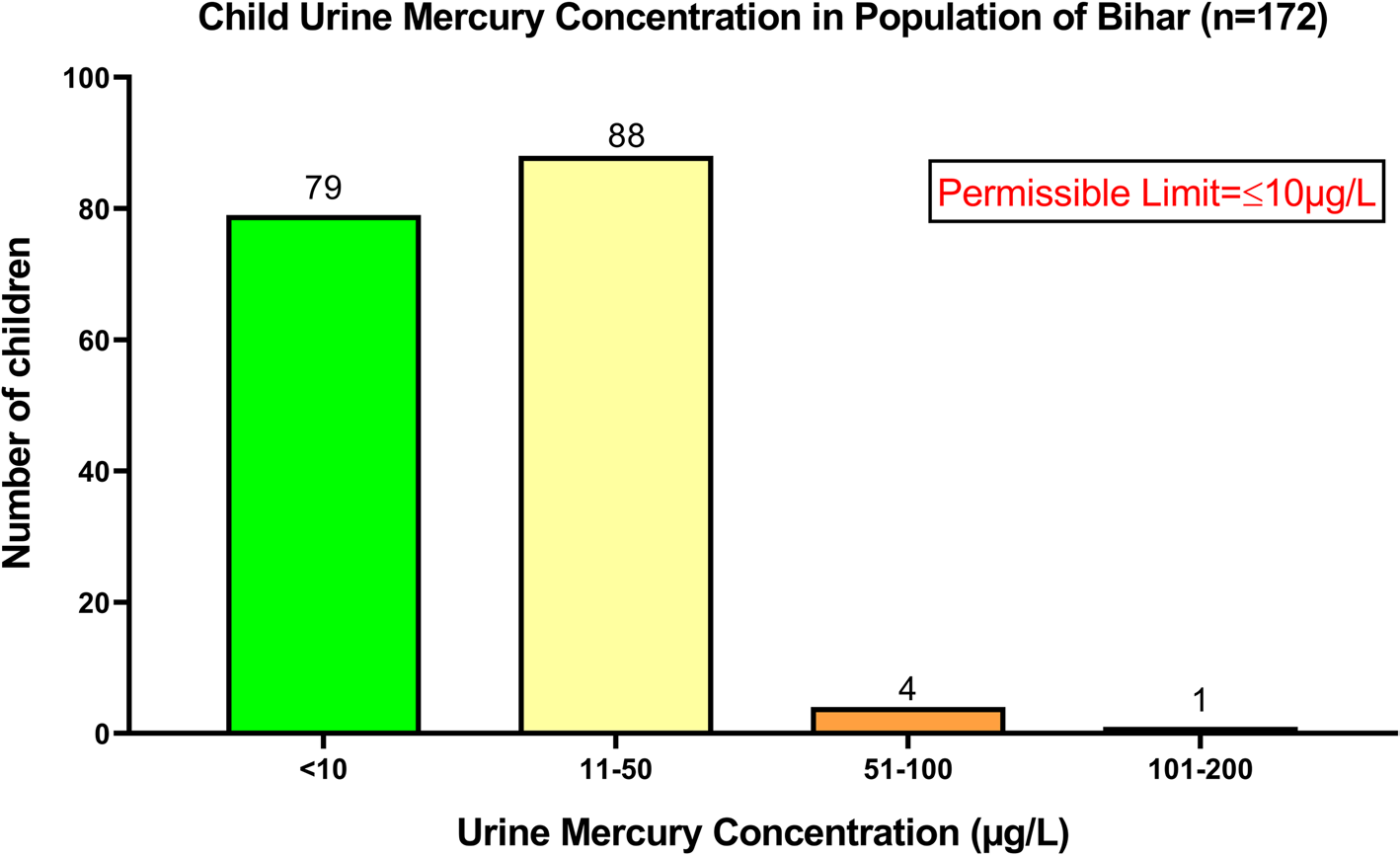
Graph showing mercury concentration in the urine samples of the studied children

*N* = 172 child subjects were approached for providing urine samples. Results indicated that *n* = 79 out of *n* = 172 child subjects, had levels of mercury in their urine samples below the permissible limit [10 µg/L], whereas *n* = 93 child subjects had the levels higher than the permissible limit. One child had the highest concentration of mercury in the urine sample as 137.56 µg/L [Fig. 5].

### Food (Wheat) mercury concentration

**Fig. 6:**
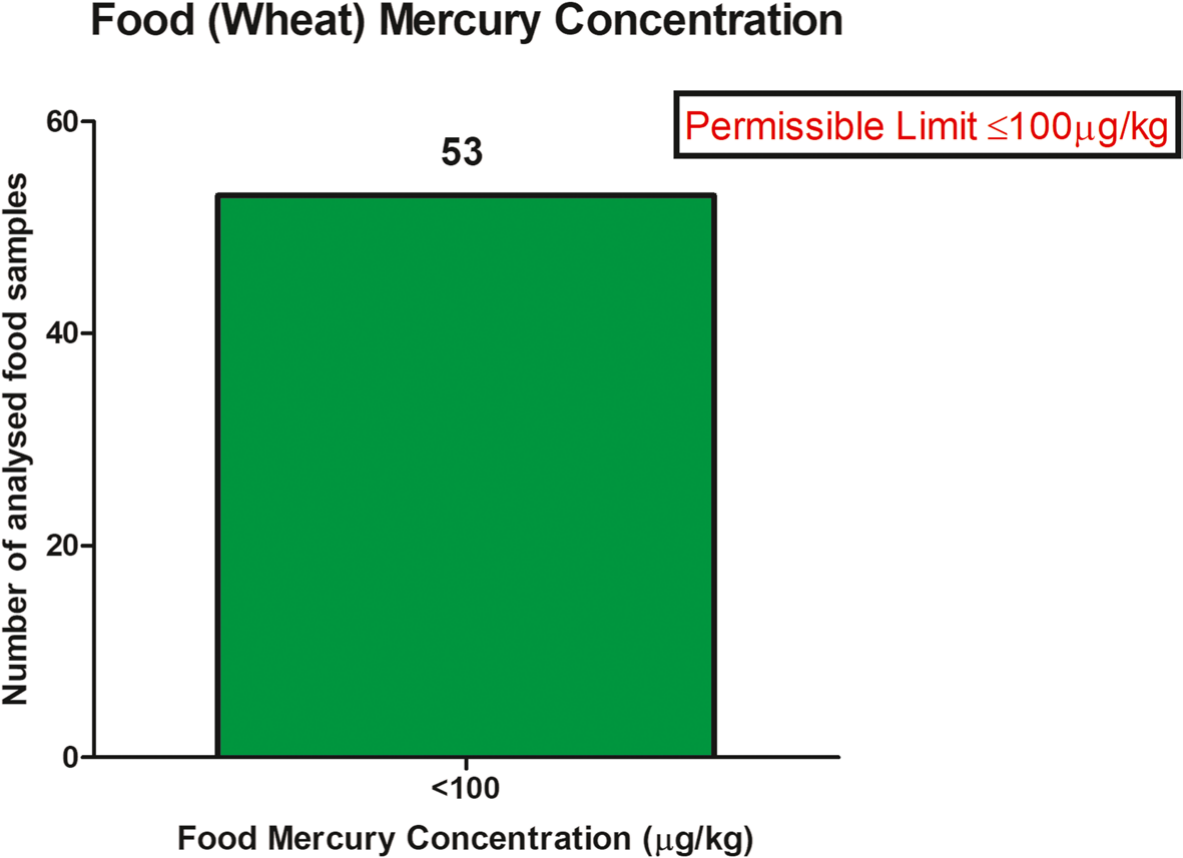
Showing food mercury concentration in studied area of Bihar

The food (wheat) samples were collected from *n*=53 households, out of *n*= 224 households while *n*= 171 households were not having the food samples. The study showed that all *n*= 53 households had their food mercury concentration below the WHO/FAO permissible level (<100 µg/Kg). The highest mercury content in the food was 44.44 µg/Kg [Figure 6].

### Multivariate analysis

**Fig. 7:**
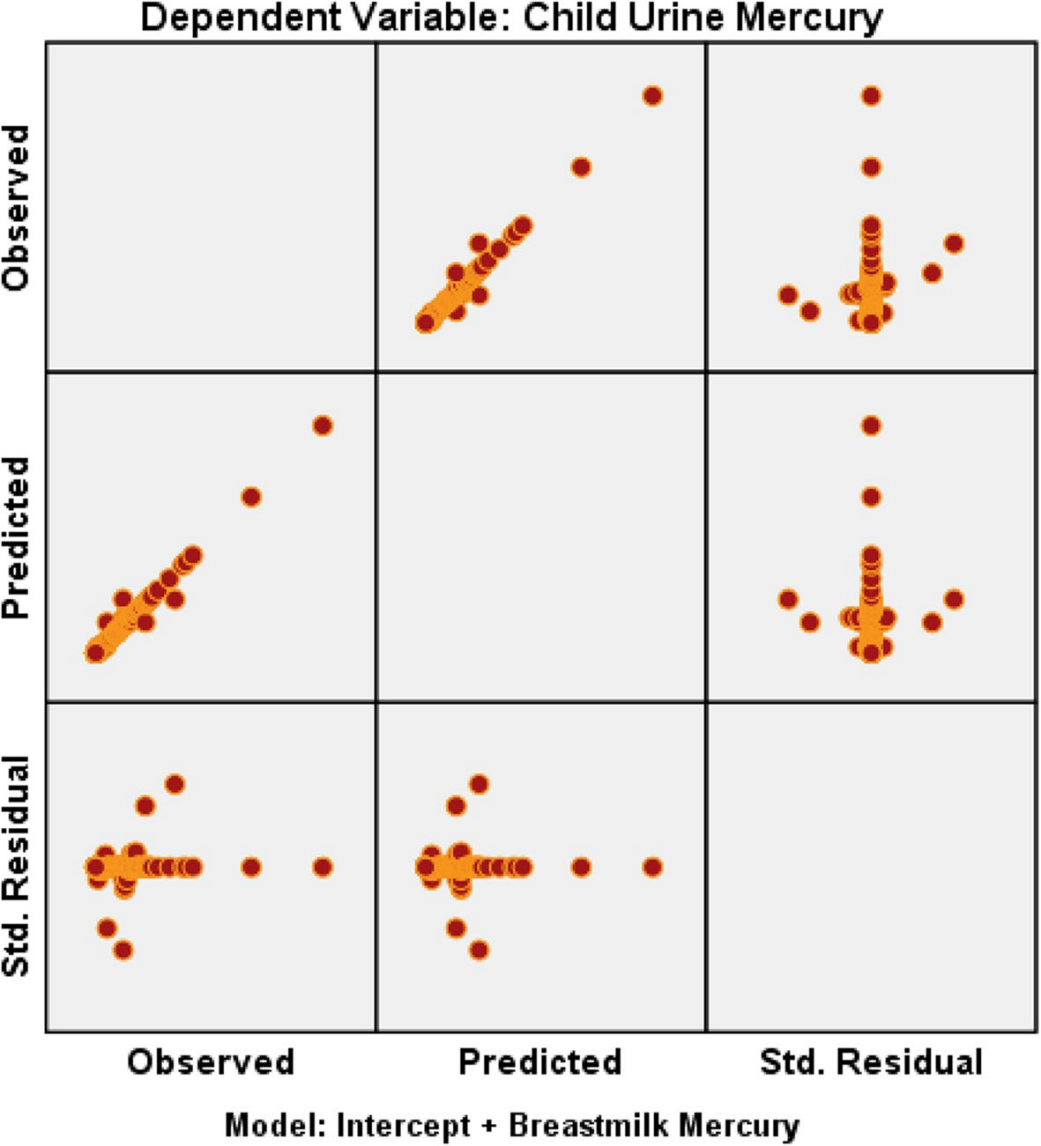
Residual plot – dependent variable—child urine mercury vs breastmilk mercury

**Fig. 8:**
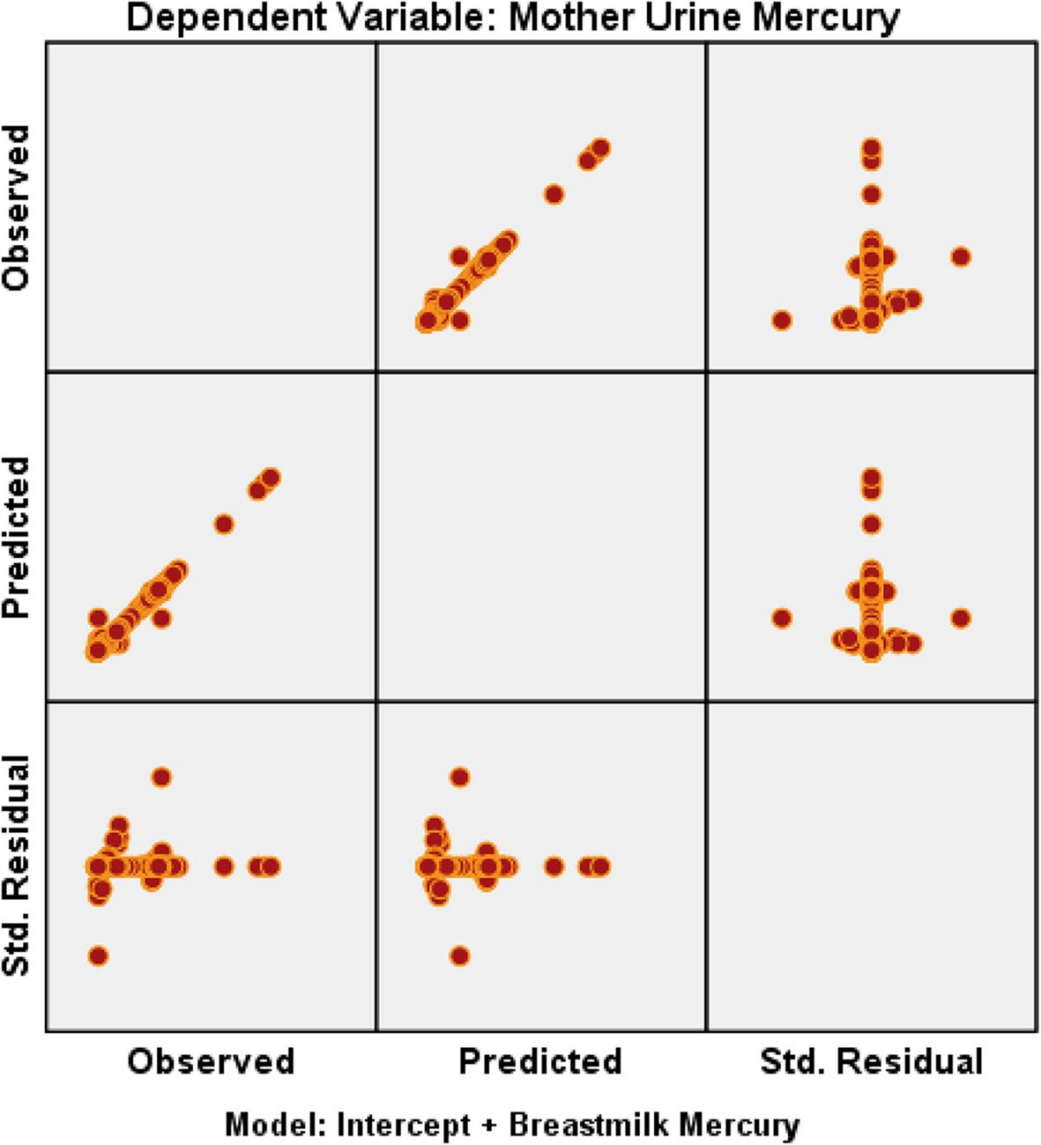
Residual plot – dependent variable – mother’s urine mercury vs breastmilk mercury

The independent variable breast mercury significantly impacts both child urine mercury and mother urine mercury. The high *R*^2^ values indicate that the model explains nearly all the variance in the dependent variables. However, the adjusted *R*^2^ values (80.6% and 82.8%) suggest some reduction in explanatory power when accounting for model complexity. The model is statistically robust, as evidenced by significant F-tests and *p*-values [Figs. 7 and 8].

### Geospatial analysis

**Fig. 9:**
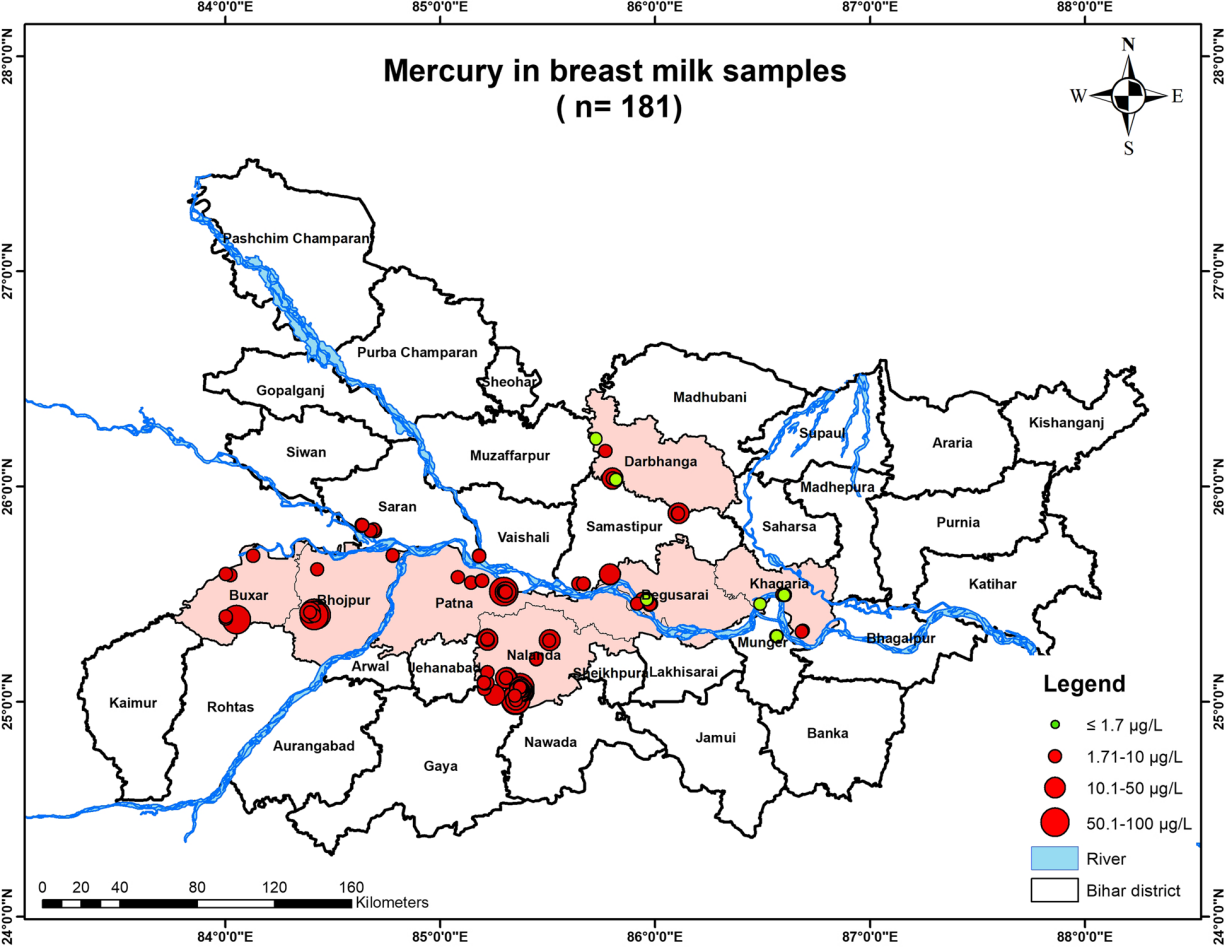
Map showing mercury contamination in the mother's breastmilk samples inhabiting in the studied districts of Bihar

**Fig. 10:**
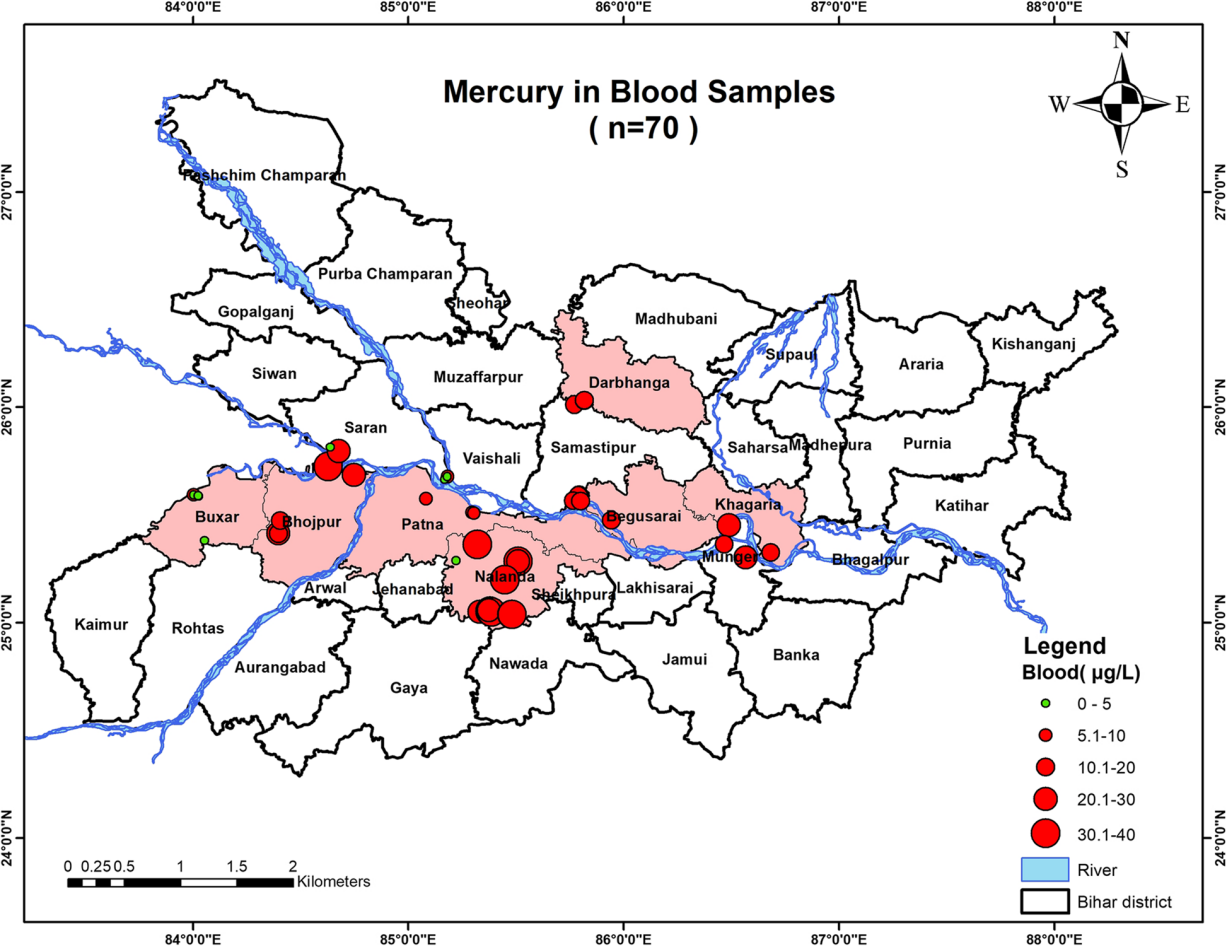
Map showing mercury contamination in the mother's blood samples inhabiting in the studied districts of Bihar

**Fig. 11:**
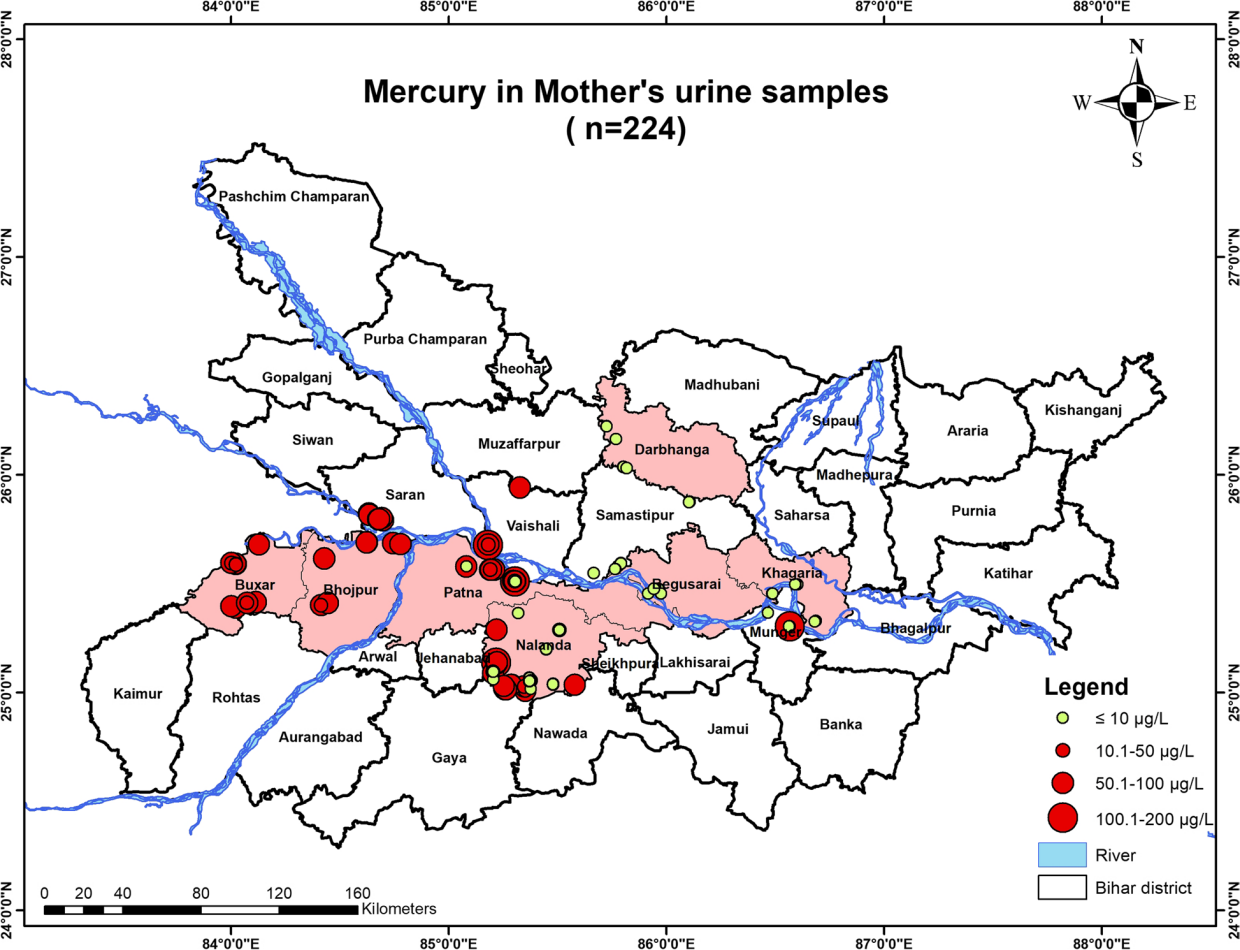
Map showing mercury contamination in the mother's urine samples inhabiting in the studied districts of Bihar

**Fig. 12:**
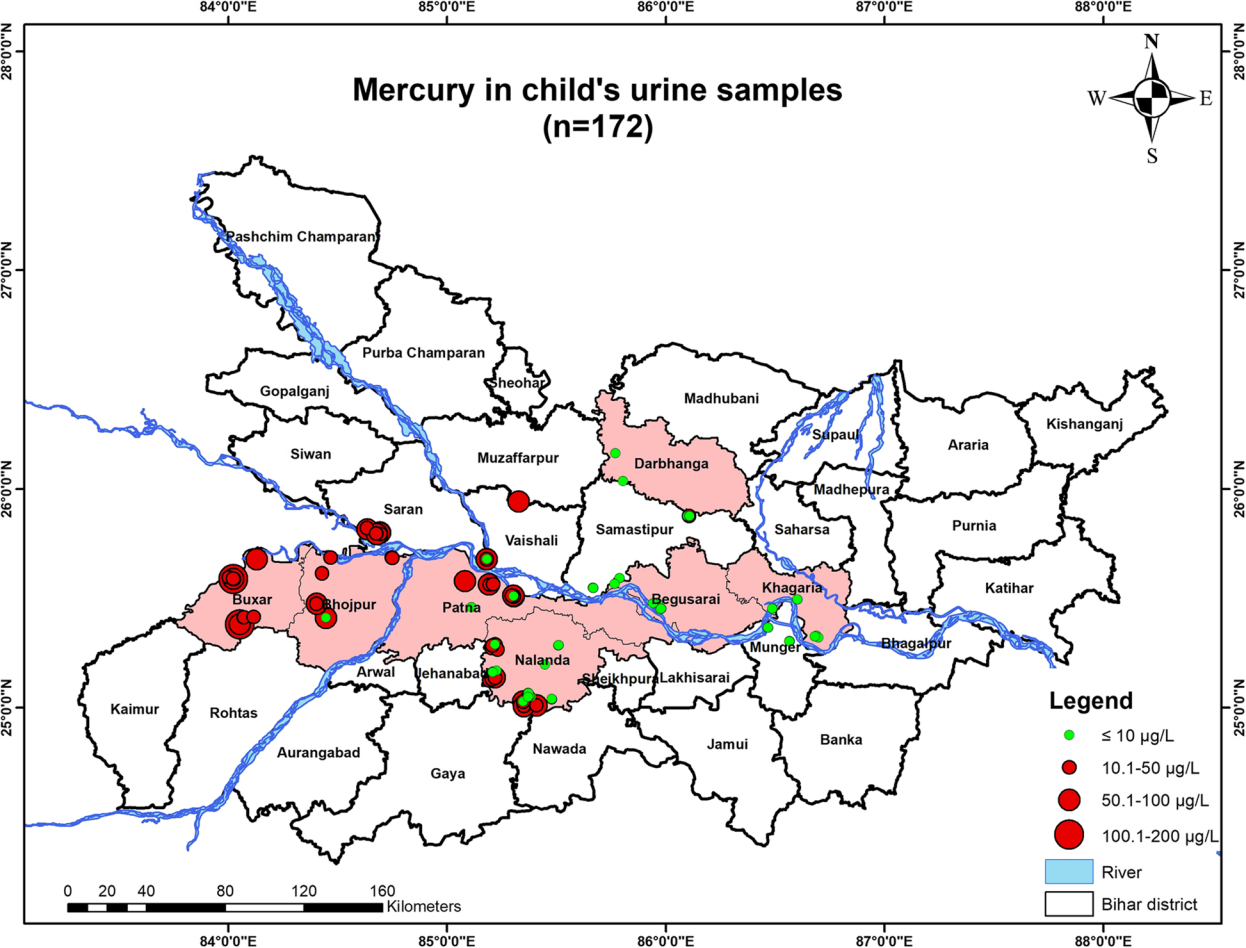
Map showing mercury contamination in the child’s urine samples inhabiting in the studied districts of Bihar

**Fig. 13:**
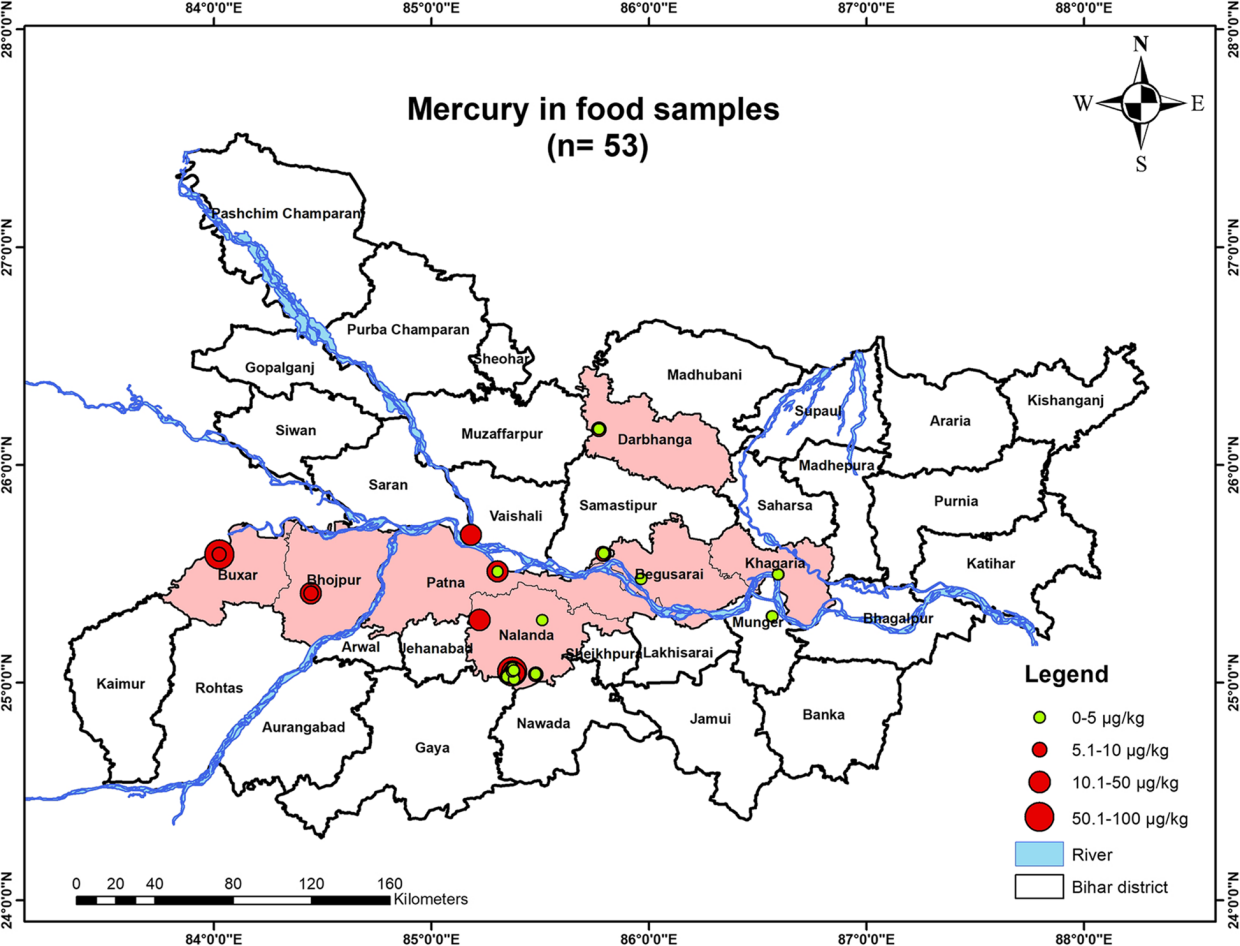
Map showing mercury contamination in the food samples consumed by the studied subjects of the exposed districts of Bihar

Using Arc-GIS Software [10.1], a shape file was created by superimposing the GPS coordinates of the samples. The primary map used was Google Maps [Google Earth]. The mercury levels were found in various bodily fluids, including breast milk, blood, and urine and were represented in µg/L. The map depicts the mercury concentration in blood, breast milk, maternal urine, and child urine along with the demographic location of their household. The study shows significant distribution of mercury contamination in the subjects inhabiting the Gangetic plains of Bihar especially Buxar, Bhojpur, Saran, Vaishali, Patna, Samastipur, Begusarai, Khagaria, Darbhanga, Munger and Nalanda districts [Figs. 9, 10, 11, 12 & 13].

### Correlation coefficient for mercury

#### Coefficient of the correlation between mercury levels in breastmilk and child’s urine

**Fig. 14:**
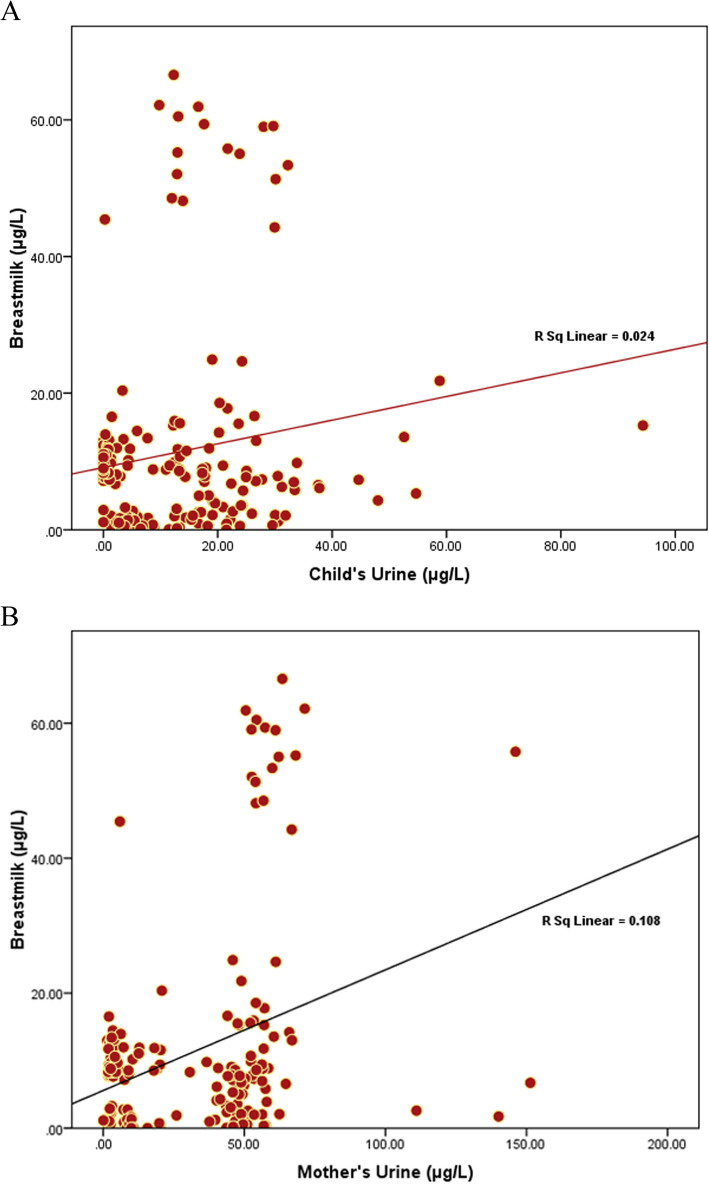
**A** Correlation coefficient study between the mercury contamination in the breastmilk and child’s urine. **B** Correlation coefficient study between the mercury contamination in the breastmilk and mother’s urine

A statistically significant correlation [*r*^2^ = 0.024 and *p* < 0.05] was observed between the mercury contamination in the breastmilk and child’s urine [Figure 14A]. The correlation shows moderate significance among the studied variables.

#### Correlation coefficient between the mercury levels in the breastmilk and mother's urine

A statistically significant correlation [*r*^2^ = 0.108 and *p* < 0.05]] was observed between the mercury contamination in the breastmilk and mother’s urine [Figure 14B].

#### Health risk assessment

**Fig. 15:**
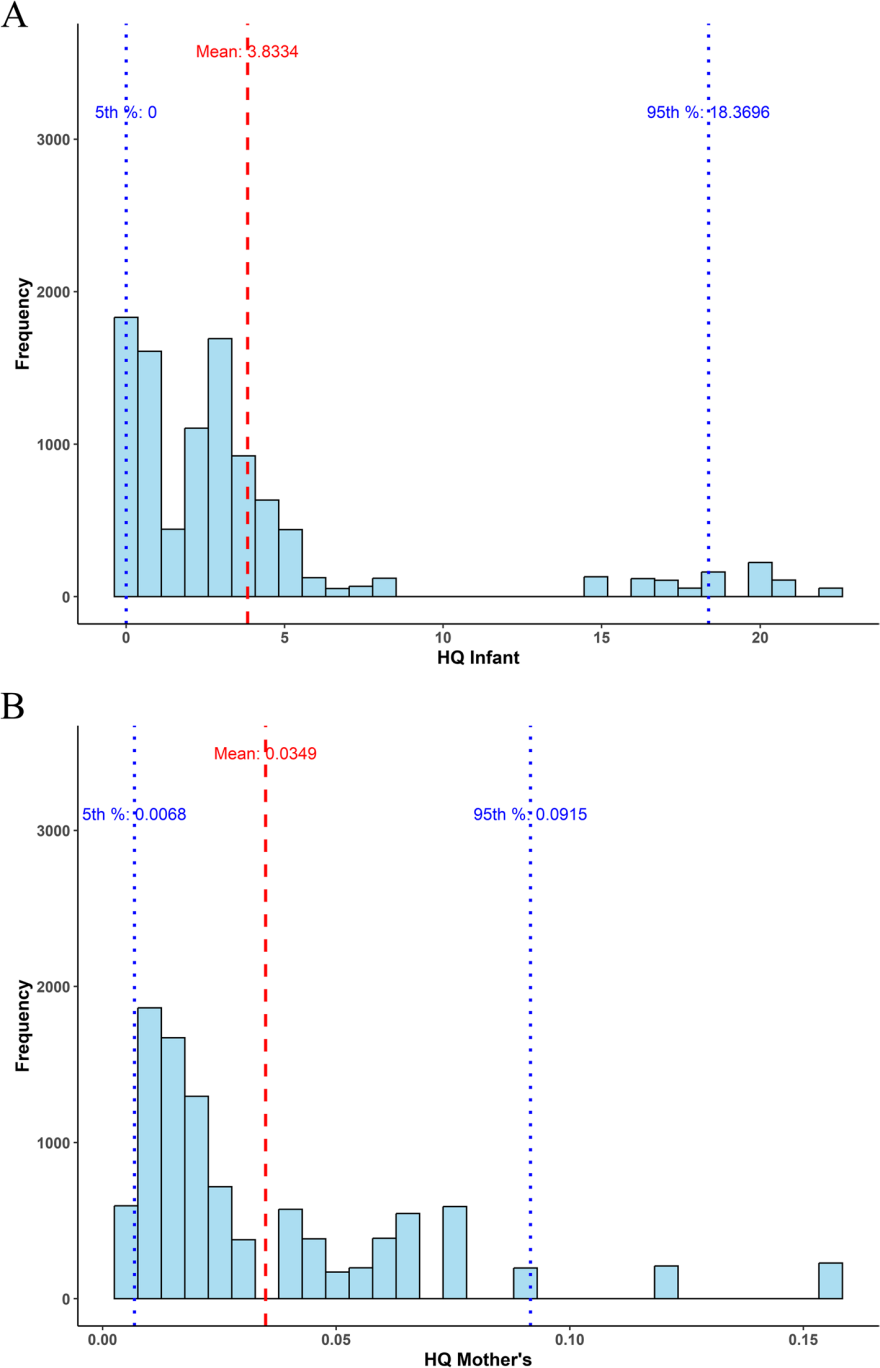
**A** Hazard Quotient of infant. **B** Hazard Quotient of lactating mother

The health risk assessment of Mother’s and their infants was calculated by using Monte Carlo stimulation (Fig. 15A & B). The health risk is represented in the form of hazard quotient. The result of the hazard quotient of infants recorded minimum value as 0, maximum value as 22.225, and mean value as 3.8334. For the hazard quotient of mother’s population, the result recorded minimum value as 0.0042, maximum value as 0.1551 and mean value of 0.0348. The minimum average daily dose (ADD) of infant is zero, while the maximum ADD was 0.0066. Similarly, the minimum ADD of lactating mother was 1.289 × 10^–6^ while the maximum ADD was 4.6533 × 10^–5^.

## Discussion

The present study depicts that 74% lactating mothers had their breastmilk highly contaminated with mercury poisoning, while 19% mothers had their blood mercury contamination more than the permissible limit. Similarly, 49% women had their mercury contamination in their urine higher than the permissible limit while their 54% infant’s urine had higher mercury contamination. This denotes that 20% of infants had the complete accumulation of mercury in their body. Moreover, in the present study contamination in the wheat samples was observed but where within the permissible limit. Still the contamination can lead to exposure at some extent. The hazardous effects of mercury make it an ideal substance that neither children nor adults should ever come into contact with it. Orün et al. [51] studied the mercury exposure in breastmilk in Turkey, in which 18% of the breastfeeding mother were lactating mercury in their breastmilk. Similarly, Yalçin et al. [52] in Turkey studied the environmental exposure of mercury in 44 lactating mothers in which the mean mercury concentration in their breastmilk was 3.42 ± 1.66 µg/L, which is still higher than the WHO permissible limit of 1.7 µg/L [53, 54]. The study also reported the presence of maternal anemia incidences which had an impact causing elevated milk mercury concentrations in the breastmilk. Olujimi et al. [55] studied the toxic effect of heavy metals on breastmilk in Ogun state of Nigeria in 37 lactating mothers and found high contamination of mercury as high as 149 ± 19 µg/L in *n* = 36 samples higher than the permissible limit. So et al. [56] reported a study carried out in US population of 56,445, with mercury estimation in the blood and urine samples. Among the US studied subjects [including children and pregnant women], the organic blood mercury elevation levels were observed in years 1999 and 2016, but levels of inorganic mercury in blood and mercury in urine had significant declination. Güngör et al. [57], studied on 29 school children and reported with mercury poisoning, in 9 subjects having higher mercury levels in their blood. Güven et al. [58] studied on 49 students of Turkey, with median blood mercury levels as 21 μg/L, with the median spot urine mercury level as 40 μg/L and the median 24-h urine mercury level as 25.8 μg/L which is higher than the permissible limit. Yüksel et al. [59] carried out a study in Turkey to investigate lead and mercury levels in maternal blood, cord blood, and placenta of pregnant women. They observed significantly higher mercury levels in maternal and fetal cord blood and lead levels in the placenta in the IUGR group. Correlation analysis suggested that higher mercury and lead levels were associated with reduced fetal growth parameters. Moreover, other studies also report the mercury poisoning in the exposed population leading to severe health hazards [60–65].

Human activities account for the vast majority of mercury emissions. One measure to limit the discharge of mercury and its toxicity to the environment is to replace it with alternatives that do not contain mercury. The biomagnification of this mercury in the food chain via the aquatic and marine food, increases the bioaccumulation in the humans who are consuming this food. Methyl mercury [MeHg], an organic form of mercury is a well-known neurotoxic environmental contaminant. In human physiology, multiple cellular factors influence MeHg-induced neurotoxicity. These factors include individual differences in tissue and cell features, the age of exposure [fetal, childhood, or maturity], and the amount of exposure. New findings validate the site-and cell-specific character of MeHg-induced neurotoxicity, as well as the role of oxidative stress in its pathophysiology [66]. The primary site of absorption of ingested methyl mercury is gastrointestinal tract [95%] whereas airborne MeHg is mainly absorbed by respiratory tract and in lesser quantity by skin [67–69]. The elemental mercury (Hg) is absorbed in gastrointestinal tract through the water or food source and enters the blood stream. It is also absorbed in the brain via blood, while most of it is eliminated through the kidneys. Moreover, the MeHg also enters the RBC via organic anion transporters [70]. Haemoglobin is the major carrier of MeHg as studies reveals the binding of MeHg to the cysteine residues of α and β chains of it. Because of its affinity to thiol group, MeHg not only binds to the hemoglobin but also conjugates with albumin, glutathione and L-cystein thus increasing water solubility [71]. This property facilitates transport of MeHg across plasma membrane and blood brain barrier [BBB]. MeHg-L-cystein conjugate mimics L-methionine [a neutral amino acid] and passes through LAT1 transporter across BBB [72]. In various organ system MeHg is demethylated through biotransformation into inorganic mercury thus both organic and inorganic mercurial species deposits are found frequently in the organisms [73, 74]. Moreover, MeHg also has the ability to damage DNA which reacts with sulfhydryl groups causing neural excitotoxicity and alterations in neurogenesis [75], increasing the oxidative stress, calcium dyshomeostasis, exacerbation of inflammation [76], and concomitantly cell death mechanisms in various tissue types [77]. The MeHg also has the devastating effects on the nervous system of infants and hampers their development when exposed to it. One of the potential mechanisms linked to mercury's neurological effects is the genotoxicity of mercurial compounds, which primarily disrupts the mitotic spindle and causes chromosomal loss. These chemicals can induce single-strand or double-strand DNA breaks as well which has been reported [78].

In India, there has been very meagre study carried out on mercury intoxication in population. This present study reports for the first time with mercury poisoning in breast milk in this eastern part of India. However, [79] & [80] have reported the arsenic and lead poisoning in the breastmilk in the Gangetic plains of Bihar [India]. Sahu et al. [81] studied and estimated that approximately 233 million individuals, including as many as 17 million individuals who resided near the 10 major hotspots in Odisha district [India], were directly or indirectly susceptible to hazardous mercury emissions. These individuals resided in and around the 10 km periphery of major industrial zones.

In the present study there was a significant correlation coefficient found between the breastmilk and child’s urine and mother’s urine. Moreover, the human health risk assessment study also correlates the mercury poisoning effect with 100% of the mother’s and 66.29% of the infants exceeding the limit of non-carcinogenic risk. The continuous mercury exposure to infants may cause toxicity symptoms such as tremors, sleeplessness, migraines, memory loss, neuromuscular effects, and cognitive and motor dysfunction are also caused due to its exposure. According to the findings, the potential health risk for infants in this research is much larger than for lactating mothers. The reason for this is that infants have a greater maximum risks quotient and an average hazard quotient that is higher. This is corroborated by the ADD data, which shows that infants are exposed to more exposure than their mothers. Similar studies have been reported by the other authors as well [82–88].

Hence, there is need of medical intervention among the mercury exposed population of Bihar where the lactating mother and their infants are at very high risk of exposure which could lead to severe health hazards. However, as for the mitigation part, there are various chelating agents available against mercury poisoning. DMPS, succimer (DMSA), and the commonly used D-penicillamine (DPCN) are among the chelation agents. Various study show that metal and inorganic mercury toxicity may be treated with DPCN, as it causes more mercury to be eliminated via the urine [89–92].

By recognizing and addressing the socio-economic implications of mercury poisoning, we can work towards protecting public health, supporting affected communities, and promoting sustainable development. For this, the problem needs to be highlighted, particularly among the vulnerable groups. It is thus crucial for both communities and policymakers to work together. For this a holistic approach is required to safeguard the population against mercury poisoning by implementing a multipronged strategy that includes environmental cleanup, medical treatment, natural remedies, and preventive measures. Moreover, addressing the problem requires a concerted effort from all stakeholders. By collective efforts a healthier environment can be created for current and future generations.

## Conclusion

The present study indicates that the habitations along the river Ganges and its tributaries, including districts like Bhojpur, Buxar, Saran, Patna, Vaishali, Samastipur, Begusarai, Khagaria, Darbhanga, Munger, and Nalanda, face severe health risks from mercury exposure. The most vulnerable group are infants, as maternal mercury levels in breast milk pose a significant risk of neurotoxicity in them. The study also implicates that the mercury content in child’s body is slowly accumulating which is deposited in vital organs which could severely harm them. The food (Wheat) is also one of the exposure routes for causing the mercury poisoning. The multivariate and the Monte Carlo analysis depicts the hazardous exposure levels in the exposed population. The geospatial study also correlates the trend of high mercury concentration observed in subjects from Nalanda, Bhojpur and Patna along the south bank of river Ganga. These regions are having anomalous mercury concentration in the studied subject’s breast milk and urine samples. The role of anthropogenic wastes cannot be ruled out and treatment of the effluents which remains unattended could also be the source of exposure. Hence, immediate medical intervention is essential to mitigate this public health concern in the exposed population.

## Supplementary Information

Supplementary Material 1

## Data Availability

Data is provided within the manuscript or supplementary information files.
