# Supplementary material for: Mercury poisoning in women and infants inhabiting the Gangetic plains of Bihar: risk assessment
Source: BMC Public Health. 2025 Apr 4;25:1275. doi: 10.1186/s12889-025-22336-9 (PMC11971891; doi:10.1186/s12889-025-22336-9)
Supplement: Supplementary file 1 — Supplementary Material 1 [file 12889_2025_22336_MOESM1_ESM.pdf]

Mahavir Cancer Sansthan &Research Centre

Phulwarisharif, Patna

Project: Assessment of Female reproductive health and child health risks in Arsenic Exposed population of Bihar

Village:-

Name: **Age:** **Sex:**

House hold Name: **Mobile No.**

Members in House hold:

Female Member Details:

Children Details:

GPS Location:

Source of Drinking Water: **Depth of HP:**

Age H.P

Disease History:

Cancer Patients in Family History:

Water Sample Collection Code:

Food Sample Collection Code:

Blood Sample Collection Code:

Urine Sample Collection Code:

Breast milk Sample Collection Code:

Hair / Nail Sample Collection Code:

Child: Saliva collection Code:

Child: Urine collection Code:

Data Collected by: **(PI Signature)**

Date:

MAHAVIR CANCER SANSTHAN

FEMALE - Clinical Details

1. **Appearance:**

   Normal ☐ **Marks of Arsenicosis** ☐ **Weight** ☐ **Age:**

2. **Any Skin Lesion**

   Palm ☐ **Sole** ☐ **Trunk** ☐ **Limbs** ☐

   Chest ☐ **Back** ☐ **Any Others** ☐

3. **Any Nodular Change**

   Area ☐

4. **Any other Problem**

   Gastritis ☐ **Anaemia** ☐ **Constipation** ☐ **Diarrhoea** ☐ **Loss of** ☐ **Appetite**

5. **Any Systemic Disease**

   Heart ☐ **Lungs** ☐ **Abdomen** ☐ **Diabetes** ☐

   BP ☐ **Others** ☐ ______________________________

6. **On Any Medication**

   Duration ☐

7. **For how much time they are using a water:**

8. **Menstrual status:**

9. **Lactation Duration:**

Data Collected by: **(PI Signature)**

CHILD - Clinical Details

MAHAVIR CANCER SANSTHAN

1. **Appearance:**

   Normal ☐ **Marks of Arsenicosis** ☐ **Age:** **Gender: M / F**

   Weight ☐

2. **Any Skin Lesion**

   Palm ☐ **Sole** ☐ **Trunk** ☐ **Limbs** ☐

   Chest ☐ **Back** ☐ **Any Others** ☐

3. **Any Nodular Change**

   Area ☐

4. **Any other Problem**

   Gastritis ☐ **Anaemia** ☐ **Constipation** ☐ **Diarrhoea** ☐ **Loss of Appetite** ☐

5. **Any Systemic Disease**

   Heart ☐ **Lungs** ☐ **Abdomen** ☐ **Diabetes** ☐

   BP ☐ **Others** ☐ ______________________________

6. **On Any Medication**

   Duration

7. **Child Details (Neurological or other problems) :**

8. **Any Development Issue:**

   Vision ☐ **Hearing** ☐ **Speaking** ☐ **Movement** ☐ **Others** ☐
